# A comparison of fMRI adaptation and multivariate pattern classification analysis in visual cortex

**DOI:** 10.1016/j.neuroimage.2009.09.066

**Published:** 2010-01-15

**Authors:** Panagiotis Sapountzis, Denis Schluppeck, Richard Bowtell, Jonathan W. Peirce

**Affiliations:** a Nottingham Visual Neuroscience, School of Psychology, University of Nottingham, Nottingham, UK; b Sir Peter Mansfield Magnetic Resonance Centre, School of Physics and Astronomy, University of Nottingham, Nottingham, UK

## Abstract

Functional magnetic resonance imaging (fMRI) has become a ubiquitous tool in cognitive neuroscience. The technique allows noninvasive measurements of cortical responses in the human brain, but only on the millimeter scale. Because a typical voxel contains many thousands of neurons with varied properties, establishing the selectivity of their responses directly is impossible. In recent years, two methods using fMRI aimed at studying the selectivity of neuronal populations on a ‘subvoxel’ scale have been heavily used. The first technique, fMRI adaptation, relies on the observation that the blood oxygen level-dependent (BOLD) response in a given voxel is reduced after prolonged presentation of a stimulus, and that this reduction is selective to the characteristics of the repeated stimuli (adapters). The second technique, multivariate pattern analysis (MVPA), makes use of multivariate statistics to recover small biases in individual voxels in their responses to different stimuli. It is thought that these biases arise due to the uneven distribution of neurons (with different properties) sampled by the many voxels in the imaged volume. These two techniques have not been compared explicitly, however, and little is known about their relative sensitivities. Here, we compared fMRI results from orientation-specific visual adaptation and orientation–classification by MVPA, using optimized experimental designs for each, and found that the multivariate pattern classification approach was more sensitive to small differences in stimulus orientation than the adaptation paradigm. Estimates of orientation selectivity obtained with the two methods were, however, very highly correlated across visual areas.

## Introduction

Functional magnetic resonance imaging (fMRI) has proven extremely useful in the noninvasive study of human brain function. Measurements of the blood oxygenation level-dependent (BOLD) signal have been used to track local increases in neural activity in a large number of studies. These include studies investigating aspects of perception, cognition, and memory. One of the limitations of fMRI, in comparison with the direct recording of neuronal responses via microelectrode, is its spatial resolution. Improvements to imaging hardware and analysis techniques have provided access to higher-resolution images at improved signal-to-noise ratios (see, e.g., Logothetis, 2008; Moon et al., 2007; Yacoub et al., 2007). However, BOLD measurements are ultimately limited in spatial resolution, because the signal is only an indirect measure of neural activity and limited by, among other things, the spatial scale of the local vascular system.

In many studies, the aim is to quantify the selectivity of clusters of neurons on a spatial scale much smaller than the 3 × 3 × 3 mm^3^ volume of a voxel used typically in current fMRI experiments. Orientation-selective cells in V1 of the primate, for example, are clustered into ‘columns’ of roughly 500 μm in diameter (Bartfeld and Grinvald, 1992; Obermayer and Blasdel, 1993). Ocular dominance columns in the human primary visual cortex have a mean width of 863 μm (Adams et al., 2007). In order to resolve differences in orientation tuning between voxels ‘traditional’, fMRI methods would require voxel dimensions considerably smaller than that of the column width.

Recent fMRI studies have demonstrated new methods for studying the selectivity of neurons in various domains (such as orientation) without requiring that the voxel size be smaller than the resolution of the ‘feature map’. These have used either selective adaptation (Grill-Spector and Malach, 2001; Krekelberg et al., 2006) or multivariate pattern analysis (MVPA) (Cox and Savoy, 2003; Haynes and Rees, 2006; Kamitani and Tong, 2005; Norman et al., 2006).

The use of adaptation has a long history in the psychophysical study of visual processing (Blakemore and Campbell, 1969; Bradley et al., 1988; Snowden and Hammett, 1996). Its use has been so prominent that it has been referred to by some as the ‘psychophysicist's electrode’. There is, however, still a debate about the exact mechanism underlying these perceptual effects (see, e.g., Desimone 1996; Grill-Spector et al., 2006).

More recently, selective adaptation effects have been demonstrated with fMRI. Adaptation can be selective for stimulus orientation (Engel, 2005; Fang et al., 2005; Larsson et al., 2006), direction of motion (Huk and Heeger, 2002; Krekelberg et al., 2005; Tolias et al., 2001), various higher-order properties of objects (Grill-Spector et al., 1999; Kourtzi and Huberle, 2005; Kourtzi et al., 2003; Sayres and Grill-Spector, 2006; Vuilleumier et al., 2002), and faces (Andrews and Ewbank, 2004; Grill-Spector and Malach, 2001; Henson et al., 2002). In general, the method relies on the observation that after prolonged or repeated presentation of a particular stimulus, the BOLD response in areas sensitive to that stimulus, is selectively reduced compared to the response to other stimuli. The methodological details of the above studies vary enormously. It is possible that the mechanisms underlying the observed reduction in BOLD signal may differ between studies and may not reflect the changes measured in psychophysics or single-unit physiology experiments. In the example of orientation selectivity, after prolonged viewing of a high-contrast grating of a particular orientation, the fMRI response to a probe of the same orientation is reduced relative to that for a differently oriented probe. The fact that adaptation is not uniform across different orientations is thought to reflect tuning in the underlying neural mechanisms.

It should be noted that there is some debate about the degree of selectivity demonstrated by the selective adaptation method in early visual areas. Boynton and Finney (2003) found no selective adaptation in V1. They suggest that this may have been caused by (a) the responses of untuned neurons in V1 and V2, (b) the fact neurons in these areas do not adapt, or (c) the fact that a low spatial frequency was used for the stimulus (0.25 cycles/°, which would result in only a fraction of a single cycle being presented to most V1 receptive fields). Fang *et al.* (2005) attribute Boynton and Finney's data to the timing of their stimulus; they found that using a prolonged adaptation period resulted in significant orientation-selective adaptation in all areas tested, although the effect was still stronger in V3 and V4. Larsson et al. (2006) used a lower-contrast probe stimulus in testing orientation selectivity and find no significant difference between the visual areas in adaptation index. The choice of probe contrast may well contribute to the previous weak selective adaptation in V1 found by Boynton and Finney (2003) and Fang et al. (2005). From electrophysiological studies in LGN (Solomon et al., 2004) and primary visual cortex of cat (e.g., Ohzawa et al., 1982, 1985) and macaque (Sclar et al., 1989), we know that adaptation causes a strong rightward shift in the contrast response curve. Due to the saturating nature of this curve, the greatest difference in response between adapted and non-adapted conditions occurs for lower contrast probes (Maffei et al., 1973). Similarly, in psychophysical studies, it has been shown that, although at detection threshold, there is a highly selective adaptation to the spatial frequency of probe– versus adapter–stimuli (Blakemore and Campbell, 1969); for higher contrast probes, the tuning of adaptation is considerably broader (Snowden and Hammett, 1996) and there is less impact on the apparent contrast of the probes following adaptation (Georgeson, 1985). The use of low-contrast probes must, of course, be traded off with the need to generate robust BOLD responses in the ROIs—the ideal stimulus is the lowest contrast for which a robust response can be measured. In this study we have followed Larsson et al. (2006) in using probes of 10% Michelson contrast.

Multivariate pattern analysis (MVPA) methods, instead, make use of small differences in the fMRI response of different voxels thought to result from small biases in the spatial distribution of the neural subpopulations sampled by each voxel. By ‘learning’ the pattern of these small biases across a large number of voxels in an independent training set, multivariate pattern analysis can successfully discriminate between stimuli in a novel set of trials. Several reports have shown that such multivariate techniques can reliably distinguish between responses to different stimuli, where more conventional, voxel-wise univariate approaches, or signal averaging across whole regions of interest could not. MVPA techniques have been used to decode the orientation of gratings (Haynes and Rees, 2005; Kamitani and Tong, 2005), direction of motion (Kamitani and Tong, 2006), and object categories (Eger et al., 2008; Haushofer et al., 2008; Haxby et al., 2001) and to study visual categorisation (Li et al., 2007) and also the encoding of global form (Ostwald et al., 2008).

It has already been demonstrated in separate studies that fMRI adaptation and multivariate techniques are capable of revealing orientation-selective responses in early visual areas. The aim of this study was to compare whether the results from the two methods are in agreement on their measurement of orientation tuning in early visual cortex. The optimal procedures for the two paradigms differ; notably, the MVPA method benefits from data acquired in a blocked design, whereas an event-related design is optimal for adaptation methods. Here we compare the two methods, each with optimal designs, for data acquired in equal periods of time. Two questions were used to frame this comparison. First, do areas that show strong orientation-specific adaptation also show high classification performance? In order to test this, we compared, for a number of visual areas, the pattern classification accuracy and selectivity of adaptation from interleaved scans in a single session. Second, we wanted to know which method was more sensitive in detecting subtle orientation differences of stimuli. To measure this, we reduced, in successive scanning sessions, the orientation difference between the two gratings in both adaptation and MVPA scans.

## Methods

### Participants

Three experienced volunteers participated in this study with written consent. Procedures were approved by the Medical School Research Ethics Committee of the University of Nottingham. Subjects participated in five scanning sessions; one session to acquire high-resolution anatomical images, one session to measure retinotopic organisation in the visual cortex, and three sessions to measure responses to gratings differing in orientation by 90° (± 45°), 50° (± 25°), and 25° (± 12.5°).

### Functional imaging

We measured blood oxygen level-dependent (BOLD) cortical responses using gradient-echo (GE) echo-planar imaging (EPI) at 3 T (Philips Achieva System, Philips Healthcare, Best, the Netherlands). The parameters for scanning were as follows: voxel size = 3 × 3 × 3 mm^3^, TR = 1.5 s, TE = 40 ms, flip angle = 75°, FOV = 192 × 192 mm^2^, 20 slices oriented perpendicular to the calcarine sulcus. To improve signal-to-noise, we acquired functional data using a pair surface receiver coils (Philips Flex-S Coils) positioned over occipital cortex.

At the beginning of each session, we obtained an anatomical image that covered the same volume as the functional images (T1-weighted MPRAGE, voxel size = 1.5 × 1.5 × 3 mm^3^). This ‘coplanar’ anatomy image was used as a proxy to register functional data to a high-resolution, whole-head anatomical image obtained in a separate session (T1-weighted 3D MPRAGE, voxel size = 1 × 1 × 1 mm^3^, 8-channel SENSE head coil) using a robust registration technique (Nestares and Heeger, 2000). We segmented the high-resolution anatomical images and generated flattened representations of the occipital cortex using standard tools (SurfRelax; Larsson, 2001).

### Definition of visual areas

Eight retinotopic regions of interest (ROIs) (V1, V2, V3, V4, V3AB, LO1, LO2, and VO1) were defined using standard phase-encoding techniques (DeYoe et al., 1996; Engel et al., 1997; Engel et al., 1994; Sereno et al., 1995). Mapping data were obtained in a separate scanning session. Areas V1, V2, and V3 have been extensively discussed in the literature (see Wandell et al., 2005 for a review). V4 was defined following Larsson and Heeger (2006). V3A and V3B are located on the dorsal side of V3 and share a common foveal representation (Press et al., 2001; Wandell et al., 2005). As the two areas cannot always be distinguished, we considered only a composite region, which we label V3AB in keeping with previous reports (Larsson et al., 2006; Montaser-Kouhsari et al., 2007). VO1 is located in the ventral occipital (VO) cortex anterior and lateral to V4 (Wandell et al., 2005). We also labeled LO1 and LO2, the two retinotopically organised regions in the lateral occipital (LO) cortex, lateral to the dorsal portion of V3 (as described by Larsson and Heeger, 2006).

### Visual stimuli and procedure

Stimuli were generated using the open-source package PsychoPy (Peirce, 2007) and were back-projected from an LCD projector at a resolution of 1024 × 768 pixels to a screen sited at the feet of the subject. To control for non-linearities in the luminance profile of the display, the screen was gamma-corrected using a psychophysical procedure of 2nd-order motion-nulling (Ledgeway and Smith, 1994). Subjects viewed the screen through prism goggles.

Stimuli consisted of oriented sinusoidal gratings (spatial frequency = 1.5 cycles/°). The gratings were presented in an annulus (inner radius = 2°, outer radius = 8°) whose edges were smoothed by a Gaussian kernel (SD of 0.083° on the inner edge, 0.333° on the outer edge). The spatial phase of the gratings was randomised every 6 frames (100 ms) to prevent retinal afterimages. Grating orientations were ± 45° (session 1), ± 25° (session 2), and ± 12.5° (session 3).

**Fig. 1:**
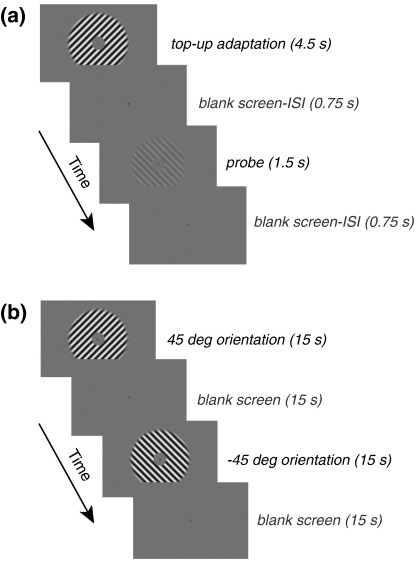
(a) An event-related design was used to measure the degree of selective adaptation, with a prolonged presentation of an adapting high-contrast stimulus, followed by a brief, low-contrast probe. (b) A block design was used to measure responses for pattern classification.

The degree of orientation-selective adaptation and the performance of the pattern classification algorithm were determined from separate, interleaved scans in the same session. An event-related design was used to measure the degree of selective adaptation. An adapting high-contrast stimulus was presented for a prolonged period, followed by a brief, low-contrast probe. A block design was used to measure the performance of the MVPA (Fig. 1).

At the beginning of each functional scanning session, we ran a localiser scan. This was followed by four adaptation scans, and three MVPA scans, which were interleaved.

#### Localiser scan

The purpose of the localiser scan was to identify voxels in the ROIs that responded to visual stimulation at the spatial location of the patterns. Stimuli were the two oriented gratings (see above) presented at high contrast (90% Michelson), alternating at 0.5 Hz for 15 s followed by presentation of a blank screen. A fixation point was present throughout. Each localiser scan consisted of 8 such blocks. The responses evoked by the localiser stimuli are available as Supplementary Data.

#### Event-related adaptation scans

The event-related fMRI adaptation protocol (Fang et al., 2005; Larsson et al., 2006; Montaser-Kouhsari et al., 2007) is shown in Fig. 1a. Participants were initially adapted to a high-contrast grating (90% Michelson) at one of the two orientations for that session for 30 s. In each subsequent trial, adaptation was maintained by presenting a ‘top-up’ adaptor for 4.5 s. There followed a blank screen for 0.75 s and the probe stimulus was then presented for 1.5 s. Probes were as follows: (a) ‘same’, a 10% Michelson grating at the orientation of the adaptor; (b) ‘different’, an equivalent grating at the other orientation for that session; and (c) a blank screen (mean luminance). These conditions were equally common and randomly chosen. Each trial ended with a 0.75 s presentation of a blank screen, giving a total duration of 7.5 s. Each scan consisted of 30 such trials (10 in each condition). In each scanning session, we ran four adaptation scans, two for each adapter orientation.

#### Block design MVPA scans

In the MVPA scans (Fig. 1b), the two oriented gratings used in the particular session (at 90% Michelson contrast) were alternated with epochs of blank screen (mean luminance) with a period 30 s (15 s ‘on’, 15 s ‘off’). Each scan consisted of 10 blocks, 5 for each orientation.

#### In both paradigms

To control for changes in the attentional state of observers, which are known to modulate fMRI responses (Brefczynski and DeYoe, 1999; Huk et al., 2001; Kastner et al., 1999; Somers et al., 1999), participants performed a demanding task at fixation. Participants were asked to count the number of target letters (X) appearing among a series of distractor letters (Z, L, N, T), which changed every 200 ms. The duration of each letter-counting trial varied randomly between 7 and 14 s. At the end of a sequence of letters, a fixation spot appeared for 1 s prompting participants to report the number of target letters presented (1–4) by pressing one of four response buttons.

### Data analysis

Functional images were motion-corrected within and between scans using MCFLIRT (Jenkinson et al., 2002). For voxels falling within different ROIs, we performed the following analyses: first, we restricted the ROI to include only voxels whose time series correlated with the stimulus epochs of the localiser scan (standard Fourier-based analysis, coherence, *c* > 0.3, phase 0 < *φ* < *π*). This ensured that the voxels included in adaptation and pattern classification analysis were selected from the same overall population but were chosen independently from either the adaptation or the MVPA measurements. We checked that our results did not depend on the exact choice of coherence threshold (analysis with *c* > 0.2 and *c* > 0.4 gave similar results).

The time series of each voxel in the restricted ROIs were then preprocessed as follows: we subtracted and divided by the mean time series to convert data from arbitrary image intensity to units of percent signal change. Responses were then filtered using a high-pass boxcar kernel (cutoff frequency, 10 cycles/scan) to remove the low-frequency drift typical in fMRI measurements (Biswal et al., 1995, 1997a,b; Purdon and Weisskoff, 1998; Smith et al., 1999; Zarahn et al., 1997).

The event-related time courses were then averaged across all voxels within the restricted ROI. Additional band-pass filtering was then applied to the averaged time courses to remove high-frequency noise and the remaining low-frequency drift (cutoff frequencies, 0.015 and 0.15 Hz). Responses to individual trials were extracted from the average ROI time course by selecting an 18 s window starting 3 s after the onset of the adaptor. The average response to the blank trials (which captures the response to the adaptor alone) was subtracted from each trial. Trials of each type were then averaged, and the resulting event-related time courses were adjusted to zero baseline.

### Adaptation index

To compute a metric describing the amount of adaptation in each ROI, we fitted event-related responses with a difference of two gamma functions (Glover, 1999; Jezzard and Clare, 2001). The amount of adaptation in each ROI was computed as the difference in the maximum values of the fitted curves (for ‘same’ and ‘different’ conditions) normalized by their sum.

$$H(t)={\left(\frac{t}{d_{1}}\right)}^{a_{1}}\exp\left(\frac{-(t-d_{1})}{b_{1}}\right)-c{\left(\frac{t}{d_{2}}\right)}^{a_{2}}\exp\left(\frac{-(t-d_{2})}{b_{2}}\right)$$

where *d*_*i*_ = *a_i_b_i_* defines the time-to-peak. The initial parameters for nonlinear regression were *a*_1_ = 5.15, *a*_2_ = 12.26, *b*_1_ = 0.97 s, *b*_2_ = 0.94 s, *c* = 0.09.

Furthermore, following Larsson et al. (2006), we assessed the statistical reliability of adaptation by computing the response amplitude of each trial. For this analysis, we first computed a mean response vector $\overset{―}{R}$ by averaging the responses for all trials regardless of the trial type,

$$\overset{―}{R}=\frac{1}{N}\sum_{i=1}^{N}R_{i},$$

where *N* is the number of trials and *R*_*i*_ is the individual trials after subtracting the response to the blank probe. Then, for each trial, we computed a scalar response amplitude *A*_*i*_ as,

$$A_{i}=\frac{R\times \overset{―}{R}}{\left||\overset{―}{R}|\right|}.$$

As in previous adaptation studies (e.g., Larsson et al., 2006), we estimated statistical reliability for individual subjects using a one-tailed *t*-test. A significant result would indicate the response amplitudes *A*_*i*_ to the probe that had a ‘different’ orientation to the adapter were significantly greater than responses to probes that had the ‘same’ orientation as the adaptor.

### Pattern classification

Classification performance depends on the number and choice of the voxels included in the analysis (Cox and Savoy, 2003; Ku et al., 2008). For each voxel in our ROIs, we determined the stimulus-driven response in the localiser scan, computed as a *t*-statistic. Following Haynes & Rees (2005), we selected an unbiased sample of 100 voxels with the highest *t*-values (stimulus versus blank) for further analysis. To quantify the dependence of classification performance on the number of voxels used, we calculated the MVPA accuracy score on 100 permutations. In each permutation, the order of the voxels included in the analysis was shuffled and the MVPA analysis was performed as described below. The mean and standard deviation of 100 of these reshuffles were then computed.

In each scanning session, we obtained data in 30 blocks (3 blocked scans, 10 blocks per scan). From each block we extracted the responses at 10 separate time points (over 15 s), delayed by three TRs to account for the haemodynamic lag. There were therefore a total of 300 time points (or repeated ‘examples’ of each response) for each voxel.

The responses of the 100 voxels at the 300 time points were sorted in a *d*-by-*n* matrix, where *d* = 100 is the number of features (voxels) and *n* = 300 the number of examples (time points). Each column of this matrix corresponds to a feature vector ***x***, which, prior to classification, was normalised to unit Euclidean length. We used a linear discriminant analysis algorithm (Duda et al., 2001) and assumed that patterns of responses recorded under the two conditions fall in multidimensional, normally distributed clusters with equal covariances. We computed the pooled covariance matrix as ∑ = (∑_1_ + ∑_2_) / 2, where ∑_1_ and ∑_2_ are the individual covariance matrices, describing the spread of each cluster. As both conditions had equal prior probabilities, a minimum-error-rate classification can be achieved by use of the linear discriminant functions:

$$g_{i}(x)=\Sigma^{-1}\mu_{i}x-\frac{1}{2}{\mu \prime }_{i}\Sigma^{-1}\mu_{i},$$

where *μ*_1_ and *μ*_2_ are the means of the two clusters. As the discriminant functions are linear, the resulting decision boundary in this two-category case is a hyperplane lying halfway between the means of the clusters.

Trials were divided into 15 groups, each group corresponding to responses collected in a pair of blocks, 1 from each orientation of the original dataset. Data from 14 of these groups were assigned to a training set and the remaining to a test set. During the training stage, the classifier learned to discriminate between responses recorded under the two orientations and to define a decision boundary. Responses from the test sample were then used to assess the performance of the classification algorithm and compute the error. Classification error was evaluated using a cross-validation procedure (Duda et al., 2001) computed as the mean across 15 leave-one-out permutations.

**Fig. 2:**
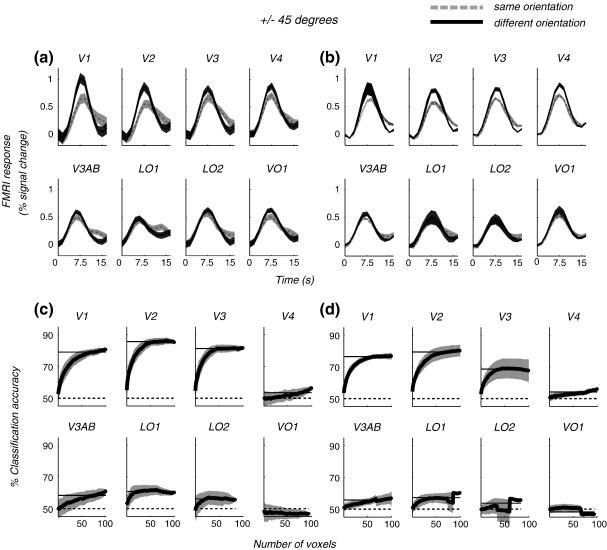
(a) Event-related modulations in fMRI signal during the adaptation sequence for one subject (JWP) and (b) averaged across subjects. The black line indicates the ‘same’ orientation condition; the gray line shows the ‘different’ orientation. Responses are averaged over 40 trials for each condition. The response to the blank condition was subtracted to account for the response to the adapting stimulus. (c) MVPA performance versus number of voxels included in the analysis for one subject (JWP). Error bars are standard deviations computed over 100 reshuffles. (d) MVPA performance versus the number of voxels averaged across subjects. Error bars represent ± 1 SEM across subjects. The dashed line shows classification accuracy based on chance (50%). The gray solid line indicates the index used to estimate classification performance (see Methods).

To compute a single metric describing the classification performance in each ROI, we computed the mean accuracy at all points between the 50th and 100th voxels. This typically captures the asymptotic performance (e.g., V2; Fig. 2c) and provides a reasonable aggregate for nonasymptotic cases (e.g., V4; Fig. 2c).

#### Permutation test for classification accuracy

To assess the statistical reliability of the multivariate classification performance, we performed a permutation test (Efron and Tibshirani, 1993). To simulate the distribution of expected classification accuracy scores under the null hypothesis, we calculated the MVPA accuracy score on 5000 resamples. Each resample was generated by shuffling the indices assigning the responses to the two different orientation conditions and performing the analysis exactly as described above. From the distribution of the classification accuracy values resulting from these resampled analyses, we obtained the 95% confidence interval for chance performance.

## Results

### Comparison of results across visual areas

We sought to examine the relationship between orientation-selective adaptation and multivariate pattern classification analysis (MVPA) across visual areas, by considering the responses to two gratings with a large orientation difference (± 45°). FMRI adaptation and MVPA were evaluated using an event-related and a blocked design, respectively (Fig. 1), which were carried out in interleaved order in a single scanning session. The probe-related modulations in fMRI signal during the adaptation sequence are shown for one subject (JWP; Fig. 2a) and averaged across participants (Fig. 2b) for eight retinotopically defined areas. The degree of selective adaptation for each area can be seen as the difference between the responses to the probe that had the ‘same’ orientation (shown in light gray) as the adapter, versus the ‘different’ orientation (dark gray). This adaptation effect is thought to reflect orientation selectivity. There was a substantial difference between the responses to the two conditions in ‘lower’ visual areas (V1, V2, V3, and V4). The adaptation appears less selective in ‘higher’ order areas (V3AB, LO1, LO2, and VO1).

Figs. 2c and d plot the performance of the pattern classifier based on linear discriminant analysis for the same visual areas in one subject (c) and across subjects (d). Classification accuracy is plotted against the number of voxels included in the analysis (see Methods). In areas V1, V2, and V3, even when classification is computed only for the single voxel, we found classification accuracy on average to be close to 70%. In these areas, classification performance increased monotonically as more voxels were included in the analysis and reached an asymptote after 10–20 voxels. In higher visual regions V4, V3AB, and LO1, classification accuracy was lower but significantly greater than chance (based on a permutation test, see Methods). The results of adaptation and MVPA for all subjects are summarised in Table 1.

**Fig. 3:**
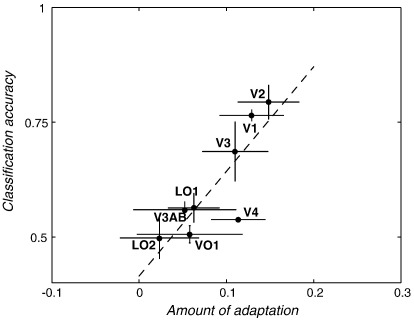
Classification accuracy plotted against the amount of adaptation across visual areas. Data are from three subjects. Error bars represent ± 1 SEM. The dashed line indicates the regression line (*r* = 0.85, *n* = 8, *p* < 0.05) computed from the averaged data across subjects.

For each method, a single selectivity index was determined; the contrast between ‘same’ and ‘different’ orientations for the adaptation study and an aggregated performance index for the MVPA (see Methods). Fig. 3 compares these selectivity indices directly in eight retinotopic visual areas averaged across the three participants. There was a strong correlation (*r* = 0.85, *n* = 8, *p* < 0.05) between classification accuracy and the selective adaptation across regions. Area V4 deviated somewhat from this pattern; it had a greater selectivity to orientation, as measured by the selective adaptation index, than would have been predicted by the MVPA performance. The V4 data point does, however, fall within the area of 95% confidence from the regression analysis, and so its reliability is unclear.

### Comparison of results with decreasing orientation differences

Next, we tested the sensitivity of adaptation and MVPA across a range of orientation differences, for areas that showed strong orientation selectivity. For this purpose, we ran two additional scanning sessions with exactly the same procedure as above, but with smaller separation between grating orientations. In one session, we tested the responses to ± 25° oriented gratings and in another to ± 12.5° gratings. As the separation between orientations is reduced, one would expect a drop in selective adaptation as well as lower classification accuracy. In the limit, this must result in a failure to discriminate responses between stimulus categories.

**Fig. 4:**
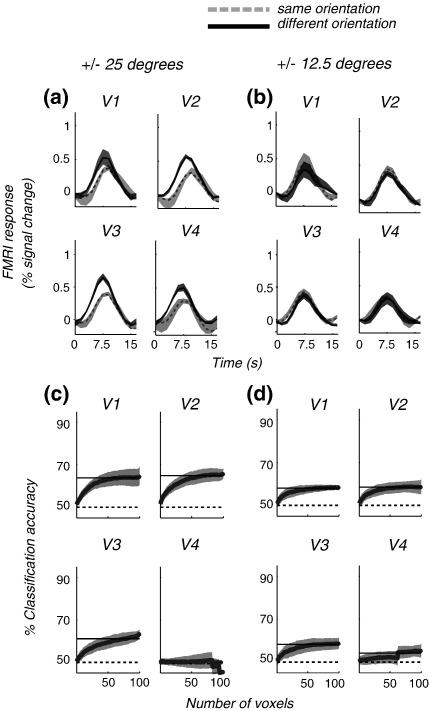
FMRI modulations in response to probe stimuli during the adaptation sequence for the (a) ± 25° and (b) ± 12.5° conditions. Performance of pattern classification versus number of voxels for the (c) ± 25° and (d) ± 12.5° conditions. Data are shown for the group average. Shaded regions represent ± 1 SEM across subjects. Same conventions as in Fig. 2.

The probe-related fMRI signal modulations are shown for the group average, for the ± 25° (Fig. 4a), and the ± 12.5° (Fig. 4b) pairs of orientations. There was still a robust selective adaptation for orientations of ± 25°. However, when separation between orientations was decreased even further (to ± 12.5°), the adaptation was not sufficiently selective to reveal any difference between the two probes. MVPA performance is also shown for the ± 25° (Fig. 4c) and the ± 12.5° (Fig. 4d) oriented gratings. As the difference in orientation between target stimuli was decreased, classification accuracy also decreased but remained above chance performance even at the smallest separation (see also Table 1).

**Fig. 5:**
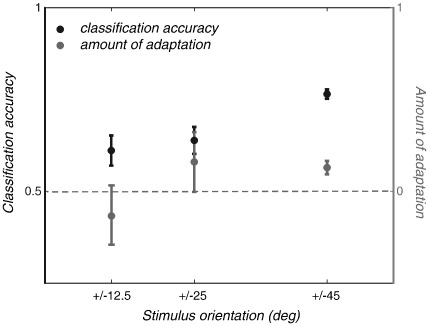
Amount of adaptation (gray) and classification performance (black) plotted against separation in stimulus orientation. Data are V1 responses averaged across three subjects. Error bars represent ± 1 SEM.

Fig. 5 shows a summary of the data for area V1. Adaptation indices and MVPA accuracy scores were averaged across participants. Note that, since the chance level for MVPA accuracy is 0.5, and the selective adaptation index should be 0 at chance, these metrics are plotted on separate *Y* axes (for both, 1.0 is the maximum possible value). Surprisingly, the ± 25° orientations did not cause any less selective adaptation than the ± 45° condition (Fig. 2a), although it fell to zero as separation became smaller (± 12.5°). The effect was also more variable between subjects at lower separations (note the size of the error bars). In contrast, classification accuracy (shown in black) falls monotonically as separation decreases, but was remarkably consistent between subjects and remained above chance performance even for small differences between target stimuli.

## Discussion

A wide range of fMRI studies have used selective adaptation or multivariate pattern classification analysis (MVPA) methods to show the selectivity of neurons on a subvoxel scale. However, it is not known whether the two methods provide consistent results about the properties of the cortical areas under study, nor is it known which technique is more sensitive. To address these questions, we compared the two methods directly for their ability to detect the well-documented orientation selectivity in early visual cortex. First, we considered results obtained with the two techniques using stimuli with large orientation differences. Second, we reduced the difference in stimulus orientations to determine the dependence of each technique on orientation differences.

Both methods were clearly capable of revealing orientation selectivity in early visual areas (V1, V2, V3). For the MVPA method, this has been shown previously by several studies using both support vector machine (Kamitani and Tong, 2005) and linear discriminant analysis (Haynes and Rees, 2005) and the pattern of results has been rather robust between groups. Kamitani and Tong found a diminishing trend of orientation preference across V1–V4, but no selectivity in + MT. Similarly, Haynes and Rees obtained higher classification accuracy in V1 than in V2 and V3.

For adaptation a number of groups have also shown that orientation selectivity can be demonstrated but the pattern of data has been more variable and seems critically dependent on the choice of experimental parameters. In particular, the duration of the adaptation period has a clear effect, with short durations failing to show orientation selectivity in area V1 (Boynton and Finney, 2003). Fang et al. (2005) used both short- and long-duration adaptation periods and show that with prolonged adaptation, the effects in V1 become measurable, although they also found greater effects in extrastriate cortex. Larsson et al. (2006) used long periods of adaptation as well as a lower-contrast probe and found roughly equal adaptation indices across V1, V2, V3, and V4. We have used similar parameters with identical findings.

We found less selectivity to orientation in later visual areas (V3AB, LO1, LO2, and VO1), using both MVPA and adaptation methods. The correlation between the results across visual areas was high (*r* = 0.85, *n* = 8, *p* < 0.05) indicating that the two methods are in strong agreement, at least in the domain of orientation specificity. This may not have been the case and increases our confidence in both methods. It also potentially informs our understanding of orientation selectivity in the areas studied. Electrophysiology studies have demonstrated that orientation selectivity is a common feature of early visual areas. This is well documented for V1 and V2, but has been less explored in V3 (for review, see Lennie, 1998). FMRI adaptation experiments had suggested a degree of selectivity, but this may have been simply a result of adaptation in earlier areas, resulting in reduced input to V3 (Larsson et al., 2006). The fact that we find high MVPA performance, as well as orientation-selective adaptation in this area, increases the confidence that human V3 does indeed code for stimulus orientation. Area V4 deviated furthest from the regression line but still fell well within its 95% confidence limits.

Similarly finding an absence of orientation-selective adaptation alone, would not rule out the presence of orientation-selective mechanisms; these may be present, but not susceptible to adaptation. This possibility is less likely when a second independent method (MVPA) also finds a lack of selectivity.

It should be noted that this pattern of results depended on our choice of parameters in the adaptation method, as discussed above. We chose to use low-contrast probes because these are known to produce robust selective adaptation effects in previous psychophysical (Georgeson, 1985; Snowden and Hammett, 1996), neurophysiological (e.g., Maffei et al, 1973), and fMRI (Larsson et al., 2006) studies. However, had we chosen a shorter adaptation period or higher-contrast probes, the correlation between the methods may have been weaker.

In order to determine how each technique depended on stimulus orientation, we performed the experiment with smaller orientation differences. MVPA performance remained above chance for all pairs of orientations tested and was remarkably consistent between participants. Selective adaptation failed to distinguish stimuli with smaller separations; for a 50° separation, it failed in one of the three individuals; for a 25° separation, it failed in all participants. This is in agreement with previous data from Fang et al*.* (2005). Their data show that, although an adaptation effect can be measured to probe stimuli as little as 7.5° from that of the adapter, the effect is not selective; the responses to such probes are statistically indistinguishable from that to probes matching the adapter in orientation. Since we are interested in the degree to which these methods can separate the underlying neuronal populations, we consider this a failure of the method at this orientation difference.

It should be noted that these results may not be mirrored in other domains of visual selectivity. For instance, measuring the degree of selectivity to spatial frequency, direction of motion or faces may give very different results if the neurons that code these dimensions in a particular area adapt strongly, but are only weakly clustered. Clearly, however, in the case of orientation selectivity measurements, the multivariate pattern analysis was rather more sensitive than the selective adaptation measure, although the two methods were in close agreement for most visual areas.

## Figures and Tables

**Table 1:** Response amplitude differences (in units of % fMRI signal change) and pattern classification accuracies for individual subjects by condition.

(A) Effects for ± 45° comparison in all areas				
	V1	V2	V3	V4
Adaptation				
JWP	**0.58 (0.012)**	**0.38 (0.035)**	0.27 (0.068)	**0.28 (0.039)**
DS	0.19 (0.051)	**0.23 (0.035)**	0.2 (0.068)	0.2 (0.132)
SH	**0.30 (0.041)**	**0.35 (0.013)**	**0.36 (0.020)**	**0.26 (0.024)**
MVPA				
JWP	**0.80 (0.000)**	**0.88 (0.000)**	**0.84 (0.000)**	**0.59 (0.000)**
DS	**0.75 (0.000)**	**0.82 (0.000)**	**0.67 (0.000)**	0.54 (0.068)
SH	**0.75 (0.000)**	**0.75 (0.000)**	**0.66 (0.000)**	**0.56 (0.001)**


	V3AB	LO1	LO2	VO1
Adaptation				
JWP	**0.22 (0.03)**	0.12 (0.172)	0.17 (0.093)	**0.23 (0.040)**
DS	0.002 (0.50)	0.15 (0.166)	0.01 (0.480)	− 0.07 (0.420)
SH	**0.17 (0.017)**	**0.17 (0.023)**	0.07 (0.208)	**0.19 (0.045)**
MVPA				
JWP	**0.62 (0.000)**	**0.65 (0.000)**	**0.62 (0.000)**	**0.54 (0.047)**
DS	0.54 (0.059)	0.53 (0.150)	0.46 (0.920)	0.54 (0.760)
SH	**0.57 (0.032)**	**0.60 (0.000)**	**0.58 (0.001)**	**0.55 (0.032)**


(B) Effects for ± 25° in all orientation-selective areas				
	V1	V2	V3	V4
Adaptation				
JWP	0.40 (0.120)	0.30 (0.165)	0.32 (0.100)	**0.21 (0.034)**
DS	0.00 (0.430)	0.07 (0.240)	0.08 (0.210)	− 0.06 (0.350)
SH	0.02 (0.386)	0.05 (0.260)	**0.24 (0.045)**	0.09 (0.360)
MVPA				
JWP	**0.66 (0.000)**	**0.67 (0.000)**	**0.67 (0.000)**	0.52 (0.190)
DS	**0.60 (0.000)**	**0.64 (0.000)**	**0.58 (0.000)**	0.48 (0.770)
SH	**0.71 (0.000)**	**0.71 (0.000)**	**0.65 (0.000)**	**0.57 (0.003)**


(C) Effects for ± 12.5° in all orientation-selective areas				
	V1	V2	V3	V4
Adaptation				
JWP	− 0.09 (0.400)	0.02 (0.480)	0.12 (0.26)	0.10 (0.300)
DS	0.11 (0.720)	0.01 (0.380)	0.06 (0.150)	0.03 (0.400)
SH	− 0.43 (0.020)	− 0.21 (0.060)	− 0.23 (0.010)	− 0.13 (0.790)
MVPA				
JWP	**0.62 (0.000)**	**0.65 (0.000)**	**0.65 (0.000)**	**0.57 (0.005)**
DS	**0.59 (0.000)**	**0.58 (0.001)**	**0.60 (0.000)**	**0.56 (0.017)**
SH	**0.61 (0.000)**	**0.60 (0.000)**	**0.57 (0.004)**	0.49 (0.560)

*P* values are shown in parentheses. Adaptation: *P* values were estimated using a one-tailed, unpaired-samples *t*-test (*df* = 37). MVPA: *P* values are estimated from a permutation test conducted on the data for each individual (5000 resamples, see Methods).

## Appendix A

Supplementary Fig. 1 . Retinotopic phase maps of (a) visual polar angle and (b) visual eccentricity representations overlaid on a patch of flattened cortex from the right hemisphere of one subject. The borders of eight retinotopic visual regions of interests (ROIs) are also shown. (c) Map of polar angle representation, indicating the coherence values of the BOLD responses in each voxel. (d) Coherence map of BOLD responses evoked by the localiser stimulus (phase 0 < *φ* < *π*). Values in all four panels are thresholded at coherence > 0.3. Scale bar approximately 1 cm.

Supplementary Fig. 2 . In order to maximize the similarity between methods, data were collected for the experiment in an interleaved manner, with alternating runs of adaptation and MVPA data collection. To check that this interleaving procedure did not itself affect the results, for example by cross-adapting the subject to both orientations, resulting in a weak adaptation index, we ran 3 further sessions for one participant (JWP) in the lowest orientation separation (± 12.5°). In these sessions, data for MVPA, for adapt-clockwise, and adapt-anticlockwise were collected on separate days, so that none could affect the other. The data were then analysed in exactly the same manner as in the original data, which are also presented here for comparison. The pattern of results is the same; for this orientation difference, there was no evident selective orientation (if anything there was actually a greater response in V1 to the ‘same’ orientation after adapting), but there remained a significant, albeit weaker, rate of success in discriminating the two orientations for the MVPA.

Supplementary Fig. 3 . We performed a further analysis to determine whether there was any consistent relationship between the methods on an individual voxel level; whether a voxel that adapts strongly might also be a strong driver of the MVPA classification. In our analyses, the former was characterized by the adaptation index calculated for individual voxels, the latter ultimately depended on the *t*-statistic of orientation preference for each voxel (see Methods). There is no particular reason that there should be a correlation between the methods on a voxel-wise basis, despite the fact that the methods agree in their analyses of the ROIs as a whole. The adaptation index is a relative measure of the responses to identical stimuli pre- and post-adaptation and does not necessarily depend on the amplitude of the response to that stimulus. Conversely, the MVPA is dependent on voxels differing in their actual responses to different stimuli. Data are shown from a single, representative subject, for two ROIs (V1 and V4) and for all three orientation separations. Plots show the adaptation index (from the adaptation scans) as a function of orientation preference (from the MVPA scans) for each voxel included in our analyses. Filled symbols label the subset (100 voxels) included in the MVPA analysis (based on their response during an independent localizer scan). Light gray represents voxels preferring clockwise stimuli; dark gray represents voxels preferring counterclockwise stimuli. There is no apparent relationship between the methods on a voxel-by-voxel basis.
