# Supplementary figures and images for: A comparison of fMRI adaptation and multivariate pattern classification analysis in visual cortex
Source: Neuroimage. 2010 Jan 15;49(2):1632–40. doi: 10.1016/j.neuroimage.2009.09.066 (PMC2793370; doi:10.1016/j.neuroimage.2009.09.066)

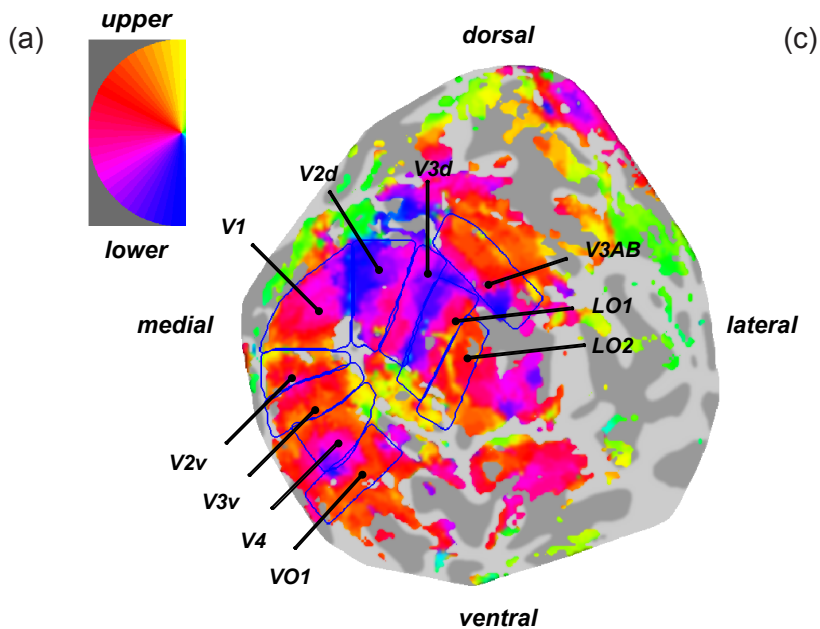

(c)

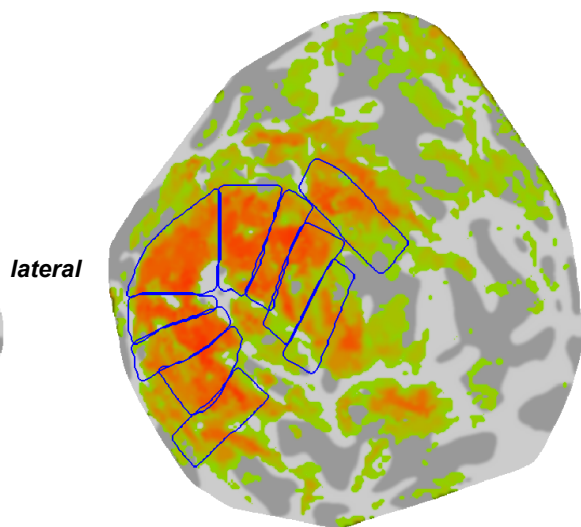

(b) *peripheral*  
*foveal*

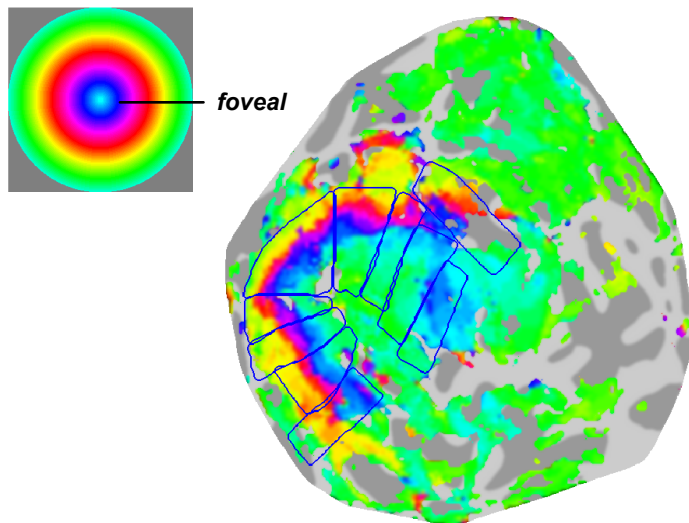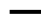

(d)

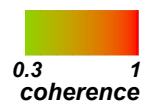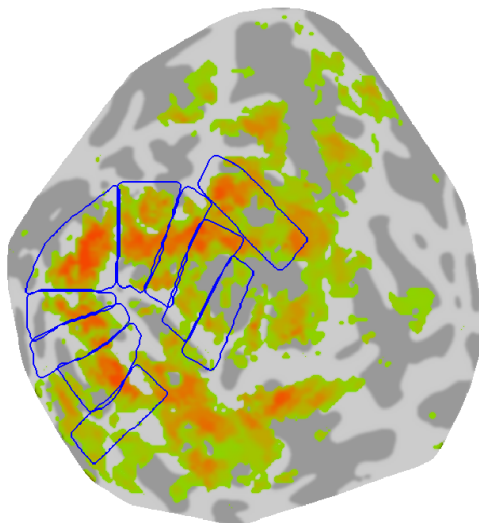

Supplement: Supplementary Fig. 1 — . Retinotopic phase maps of (a) visual polar angle and (b) visual eccentricity representations overlaid on a patch of flattened cortex from the right hemisphere of one subject. The borders of eight retinotopic visual regions of interests (ROIs) are also shown. (c) Map of polar angle representation, indicating the coherence values of the BOLD responses in each voxel. (d) Coherence map of BOLD responses evoked by the localiser stimulus (phase 0 < φ < π). Values in all four panels are thresholded at coherence > 0.3. Scale bar approximately 1 cm. [file mmc1.pdf]

--- same orientation  
— different orientation

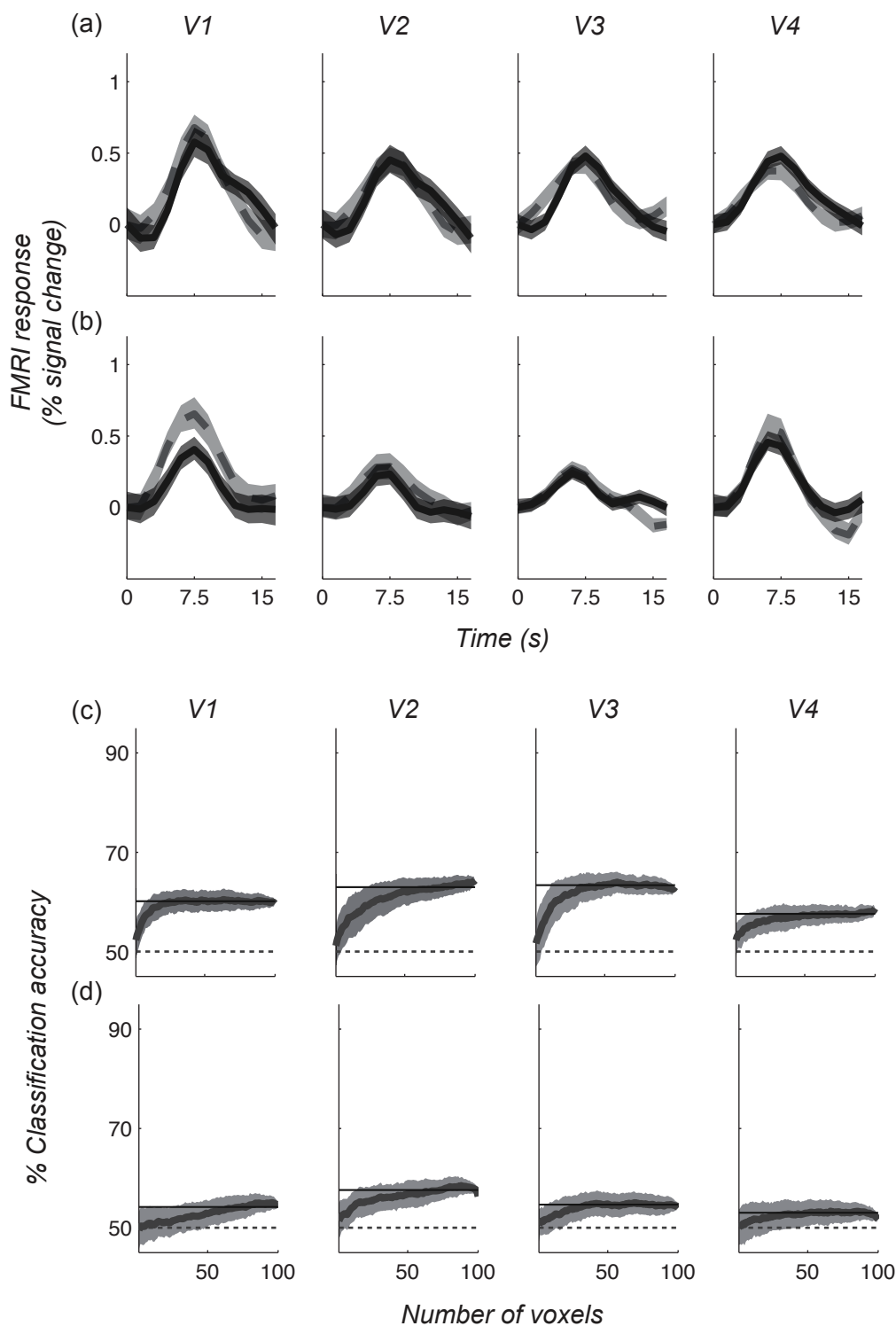

Supplement: Supplementary Fig. 2 — . In order to maximize the similarity between methods, data were collected for the experiment in an interleaved manner, with alternating runs of adaptation and MVPA data collection. To check that this interleaving procedure did not itself affect the results, for example by cross-adapting the subject to both orientations, resulting in a weak adaptation index, we ran 3 further sessions for one participant (JWP) in the lowest orientation separation (± 12.5°). In these sessions, data for MVPA, for adapt-clockwise, and adapt-anticlockwise were collected on separate days, so that none could affect the other. The data were then analysed in exactly the same manner as in the original data, which are also presented here for comparison. The pattern of results is the same; for this orientation difference, there was no evident selective orientation (if anything there was actually a greater response in V1 to the ‘same’ orientation after adapting), but there remained a significant, albeit weaker, rate of success in discriminating the two orientations for the MVPA. [file mmc2.pdf]

● *cw*-preferring  
● *ccw*-preferring

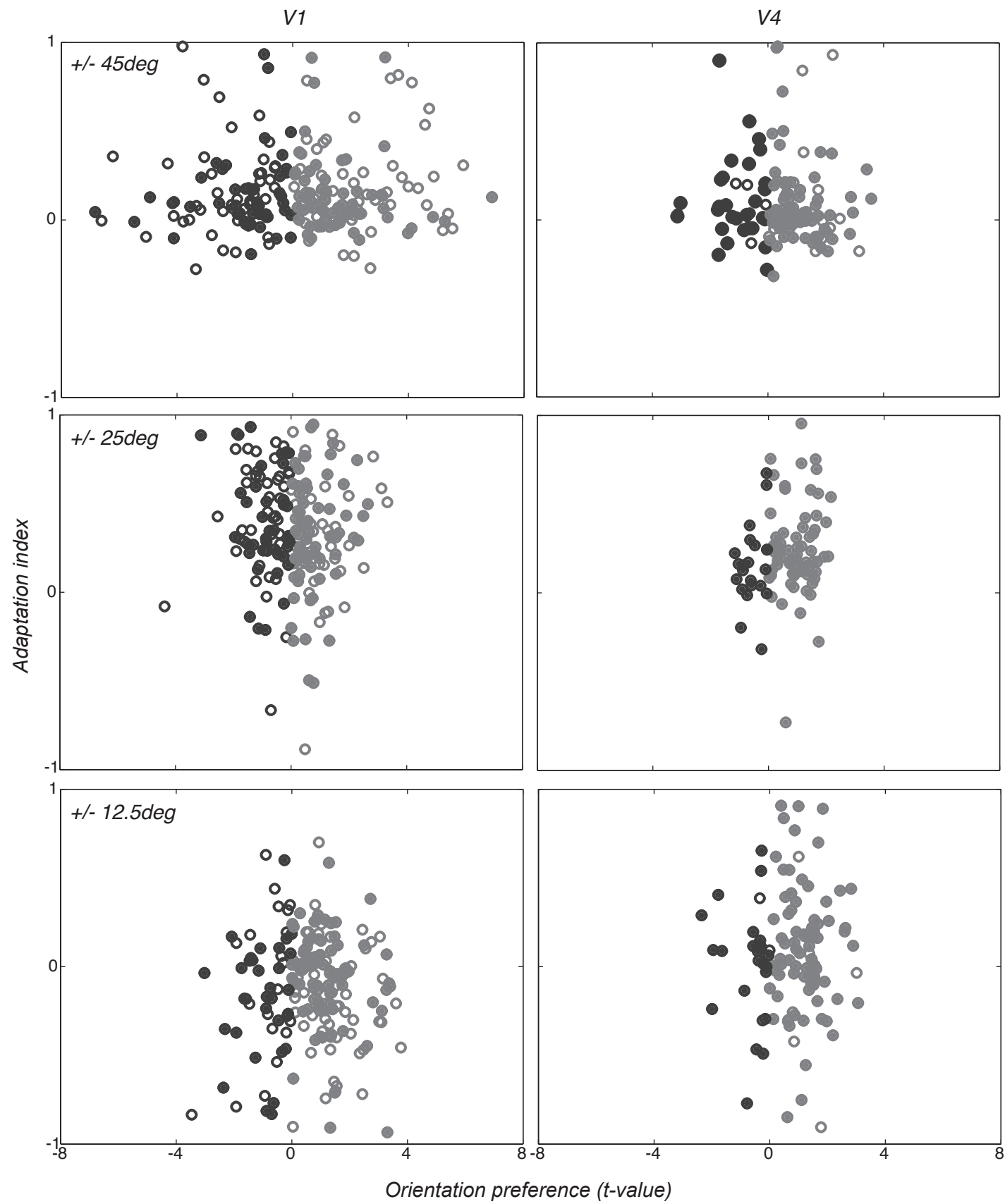

Supplement: Supplementary Fig. 3 — . We performed a further analysis to determine whether there was any consistent relationship between the methods on an individual voxel level; whether a voxel that adapts strongly might also be a strong driver of the MVPA classification. In our analyses, the former was characterized by the adaptation index calculated for individual voxels, the latter ultimately depended on the t-statistic of orientation preference for each voxel (see Methods). There is no particular reason that there should be a correlation between the methods on a voxel-wise basis, despite the fact that the methods agree in their analyses of the ROIs as a whole. The adaptation index is a relative measure of the responses to identical stimuli pre- and post-adaptation and does not necessarily depend on the amplitude of the response to that stimulus. Conversely, the MVPA is dependent on voxels differing in their actual responses to different stimuli. Data are shown from a single, representative subject, for two ROIs (V1 and V4) and for all three orientation separations. Plots show the adaptation index (from the adaptation scans) as a function of orientation preference (from the MVPA scans) for each voxel included in our analyses. Filled symbols label the subset (100 voxels) included in the MVPA analysis (based on their response during an independent localizer scan). Light gray represents voxels preferring clockwise stimuli; dark gray represents voxels preferring counterclockwise stimuli. There is no apparent relationship between the methods on a voxel-by-voxel basis. [file mmc3.pdf]
